# Role of Lysyl oxidase-like 1 gene polymorphisms in Pakistani patients with pseudoexfoliative glaucoma

**Published:** 2012-04-25

**Authors:** Shazia Micheal, Muhammad Imran Khan, Farah Akhtar, Mahmood Ali, Asifa Ahmed, Anneke I. den Hollander, Raheel Qamar

**Affiliations:** 1 Department of Biosciences, Faculty of Science, COMSATS Institute of Information Technology, Islamabad, Pakistan; 2 Department of Ophthalmology, Radboud University Nijmegen Medical Centre, Nijmegen, The Netherlands; 3 Department of Human Genetics, Radboud University Nijmegen Medical Centre, Nijmegen, The Netherlands; 4 Al-Shifa Eye Trust Hospital, Rawalpindi, Pakistan; 5 Shifa College of Medicine, Islamabad, Pakistan

## Abstract

**Purpose:**

Single nucleotide polymorphisms (SNPs) rs1048661 (p.R141L) and rs3825942 (p.G153D) in the lysyl oxidase-like 1 (*LOXL1*) gene have been previously reported to be associated with pseudoexfoliation glaucoma (PEXG) in various Asian and European populations, but these SNPs have not yet been studied in the Pakistani population. Therefore the aim of the present study was to investigate the association of these two coding *LOXL1* SNPs in Pakistani PEXG patients.

**Methods:**

One hundred twenty-eight Pakistani patients diagnosed with PEXG and 180 healthy controls were recruited for the study. Genomic DNA was extracted and both SNPs were genotyped by direct sequencing. Association of genotype and allele frequencies with PEXG were analyzed using the Chi-square (χ^2^) test.

**Results:**

Genotype and allele frequencies of both rs1048661 and rs3825942 were found to be significantly associated with PEXG. The GG genotypes of both *LOXL1* SNPs were associated with an increased risk of developing PEXG. In addition the G alleles of rs1048661 and rs3825942 confer an increased risk for PEXG with an odds ratio (OR) of 2.98 (95% CI 1.94–4.57) and OR 6.83 (95% CI 2.94–16.67), respectively.

**Conclusions:**

A significant association was found for the G allele of rs1048661 and rs3825942 in PEXG patients of Pakistani origin.

## Introduction

Pseudoexfoliative glaucoma (PEXG) occurs when pigment and abnormal basement membrane material from the anterior segment of the eye deposit in the trabecular meshwork (TM), which raises the intraocular pressure of the eye causing a degeneration of the optic nerve. In addition the presence of pseudoexfoliative material causes changes in the cornea, camerular angle, lens and zonules as well as a significant loss in the number of axons [1,2]. The accumulation of exfoliative material in the juxtacanalicular tissue (JCT) results in the disorganization of the JCT and Schlemm’s canal followed by dysfunction of endothelial cells, which appear to be the causative factor in the development of PEXG [3].

The precise etiology and pathogenesis of pseudoexfoliative glaucoma still remains unknown. Previous molecular, biochemical and immunohistochemical studies have elucidated that proteins of the extracellular matrix (ECM), metabolism and cellular stress are differentially expressed in tissues of PEXG patients. It has been observed that the pathophysiology of PEXG involves an excessive production of elastic microfibrillar components, changes in the enzymatic cross linking processes, a proteolytic imbalance between matrix metalloproteinases and their inhibitors. It is also conceivable that lack of antioxidants and increased cellular and oxidative stress may play an important role in the progression of PEXG as a stress-induced elastic microfibrillopathy [4,5]. A family of five lysyl oxidase enzymes (LOX, LOXL1, LOXL2, LOXL3, and LOXL4) play an important role in cross-linking between collagen, elastin and fibrillin, which are the substrates of the LOXL1 enzymes in the connective tissues. This cross-linking reaction provides additional mechanical strength to the ECM and makes it more resistant to degradation [6]. It has been reported in a recent study that greater cross linking in the tissues of TM and ECM makes the tissues stiffer in glaucomatous eyes as compared to the non-glaucomatous eyes [7]. LOXL1 expression is enhanced in the TM by the cytokine tumor growth factor beta1 (TGF β1). This changes the cross linking ability of LOXL1 in PEXG, which results in the deposition of exfoliative material and increased resistance in the aqueous outflow pathway [8].

A recent genome-wide association study in PEXG patients from Iceland and Sweden identified a strong association with two non-synonymous polymorphisms in *LOXL1* [9]. Similarly in German and Italian populations a strong association has been observed between PEXG and polymorphisms in *LOXL1*, which support an important role of this gene in the progression of PEXG in various populations [10]. In a meta-analysis of *LOXL1* polymorphisms in different populations including Caucasian, Japanese, Indian, and Chinese it has been observed that the G allele of rs3825942 is a common disease associated allele, with an overall odds ratio of 10.89 (95% CI 7.20–16.45). However, only in the Caucasians case the G allele of rs1048661 was found to impart an increased risk to the disease (OR 2.35) while in the Japanese population the G allele was found to have a highly protective role (OR 0.03). In the Chinese and Indian populations this polymorphism was not found to be significantly associated with PEXG [11].

Although single nucleotide polymorphisms (SNPs) in *LOXL1* have been studied in many populations, an analysis of PEXG patients from Pakistan had not yet been performed. Therefore the aim of the present study was to analyze the two non-synonymous polymorphisms (rs1048661 and rs3825942) in *LOXL1* in PEXG patients from Pakistan.

## Methods

### Patients

The present case control study included 128 patients diagnosed with PEXG and 180 healthy controls of Pakistani origin who were recruited from the Al-Shifa Eye Trust Hospital in Rawalpindi, Pakistan. Complete ophthalmic examinations were performed for both patients and controls as reported previously [12]. Briefly, clinical evaluation of patients included measurement of cup-to-disk ratio, intra-ocular pressure and slit lamp biomicroscopy to detect the presence of exfoliative material. PEXG was diagnosed in patients who presented with an accumulation of microfibrillar deposits or exfoliative material on the papillary ruff, a clear annular zone, or flakes of exfoliative material with a grayish central disc on the anterior lens capsule, iris, or corneal endothelium in one or both eyes. Controls and cases were matched for age, gender and ethnicity (Punjabi).

### Genomic DNA and genotyping

The current study was approved by the institutional review board of the Al-Shifa Eye Trust Hospital, Rawalpindi, Pakistan, and adhered to the tenants of the Declaration of Helsinki. Whole blood samples of both patients and controls were drawn by venipuncture after obtaining informed written consent. The blood samples were collected in EDTA vacutainer tubes (Becton Dickinson, Franklin Lakes, NJ) and stored at 4 °C till further processing. Genomic DNA was obtained from the whole blood using a standard phenol chloroform method [13].

Genomic DNA was genotyped for two non-synonymous SNPs located in exon 1 of *LOXL1*: rs1048661 (c.758G>T; p.Arg141Leu) and rs3825942 (c.794G>A; p.Gly153Asp). The following primers were used to amplify a 259 bp product, which spans both the polymorphic sites in exon 1 of *LOXL1*: forward primer 5′-ATT CGG CTT TGG CCA GGT GC-3′ and reverse primer 5′ GTA CAC GAA ACC CTG GTC GT-3′. The polymerase chain reaction (PCR) mixture contained 2.5U Taq polymerase (Roche, Mannheim, Germany), 1× PCR buffer, 1.5 mM MgCl_2_, 10 µM each primer and 50 ng genomic DNA. PCR amplification was performed with an initial denaturation at 95 °C for 5 min, followed by 35 cycles of denaturation at 95 °C for 45 s, primer annealing at 65 °C for 45 s, extension at 72 °C for 45 s, followed by a final extension for 7 min at 72 °C. PCR products were separated on a 2% agarose gel and purified using PCR clean-up purification plates (NucleoFast® 96 PCR; Macherey-Nagel, Duren, Germany), according to the manufacturer’s protocol. Purified PCR products were analyzed by Sanger sequencing using an automated DNA sequencer (Big Dye Terminator chemistry, version 3 on a 3730 DNA analyzer; Applied Biosystems, Inc., Foster City, CA).

### Statistical analysis

Data were analyzed using the SPSS statistical package (SPSS version 16.0; SPSS, Chicago, IL). Odds ratios (OR) for genotype and allele frequencies of patients compared to controls were calculated using the Chi-square (χ^2^) test, taking a 95% confidence interval (95% CI) into account. A p-value <0.05 was considered as statistically significant.

## Results

All PEXG patients and control individuals originated from the Punjab province of Pakistan. The PEXG patients had a mean age of 47.3±10.3 years and included 54% males and 46% females, whereas the controls had a mean age of 45.2±10.8 years with 52% males and 48% females.

**Figure 1:**
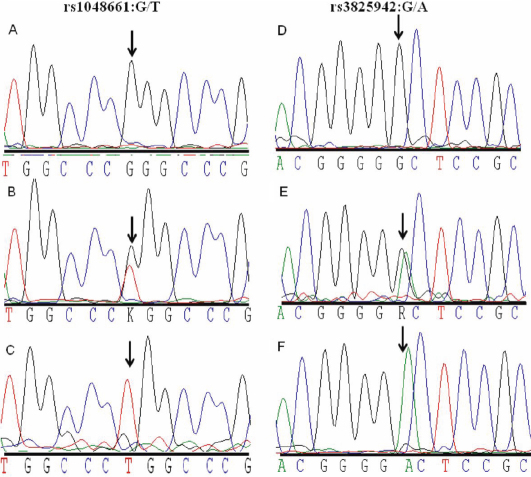
Sequence chromatograms of the two *LOXL1* SNPs of Exon 1. **A**-**C**: The corresponding normal (GG), heterozygous (GT), and homozygous variant (TT) sequences for rs1048661. **D**-**F**: Normal (GG), heterozygous (GA), and homozygous variant (AA) sequences for rs3825942.

The distribution of the *LOXL1* genotype and allele frequencies of SNPs rs1048661 and rs3825942 were analyzed in PEXG patients and control individuals. Figure 1 shows the normal, heterozygous and homozygous variant sequences for the two SNPs studied. The genotype frequencies were consistent with the Hardy–Weinberg equilibrium (HWE) for both SNPs in the controls. Table 1 gives the detailed count of the genotype and allele frequencies of rs1048661 and rs3825942 polymorphisms of *LOXL1*. For both SNPs a significant association was identified for the genotype (p-value <0.001) and allele frequencies (p-value <0.001) in PEXG patients as compared to controls. The G allele of rs1048661 was found in 85.2% of the patient alleles compared to 65.8% of control alleles, demonstrating that it confers an increased risk for PEXG (OR 2.98 [95% CI 1.94–4.57]). The G allele of rs3825942 had an even stronger association with PEXG, as it was found in 97.3% of patient alleles compared to 83.9% of control alleles (OR 6.83 [95% CI 2.94–16.67]).

**Table 1:** *LOXL1* SNPs genotype and allele frequencies in PEXG patients and controls.

rs1048661** G/T**	**Controls n=180 (%)**	**PEXG n=128 (%)**	**p-value**	**p-value**	**OR (95% CI)**
GG	78 (43.3)	91 (71.1)	0.00000064		
GT	81 (45.0)	36 (28.1)		0.0001	2.63 (1.55–4.45)
TT	21 (11.7)	1 (0.80)		0.00001	24.50 (3.37–500)
**Alleles**					
G	237 (65.8)	218 (85.2)	0.0000001		2.98 (1.94–4.57)
T	123 (34.2)	38 (14.8)			
rs3825942** G/A**	**Controls n=180 (%)**	**PEXG n=128 (%)**	**p-value**	**p-value**	**OR (95% CI)**
GG	130 (72.2)	121 (94.5)	0.000003		
GA	42 (23.3)	7 (5.50)		0.00001	5.58 (2.30–14.19)
AA	8 (4.5)	0 (0.00)		0.007	(N/A)
**Alleles**					
G	302 (83.90)	249 (97.3)	0.0000001		6.83 (2.94–16.67)
A	58 (16.10)	7 (2.70)			

## Discussion

SNPs in *LOXL1* have previously been associated with PEXG in various European and Asian populations, to date these have not been studied in the Pakistani population. The current study is the first to demonstrate that *LOXL1* SNPs are associated with PEXG patients from Pakistan. The highest association was observed with the G allele of rs3825942 (OR 6.83), which was present in an overwhelming majority of the cases with PEXG. A significant but lower association was also noted with the G allele of rs1048661 (OR 2.98).

A recent meta analysis of several studies from different populations demonstrated a strong association between PEXG and the G allele of SNP rs3825942 in Caucasian and Asian populations (overall OR 10.89), while the G allele of SNP rs1048661 was found to confer an increased risk in only the Caucasian populations (OR 2.35), whereas in the Japanese population it has a protective role (OR 0.03). This suggests that rs1048661 is not directly implicated in the disease etiology of PEXG. It is more likely that rs3825942 is the common disease-associated polymorphism and may have functional impacts on the LOXL1 protein [11]. This is in line with our findings in the Pakistani population as we found a higher OR (6.83) for rs3825942 as compared to rs1048661 (2.98). Similar results were obtained in recent studies of Latin American and Uygur populations, which were not included in the meta analysis of *LOXL1* polymorphisms [14,15].

Not surprisingly our results differ from those reported in the Indian population; the G-allele of the rs1048661 polymorphism was found less frequently in Indian cases and controls (allele frequencies of 72.1% and 63.4%, respectively) and was not found to be significantly associated with PEXG [16]. The allele frequencies in our Pakistani cases and controls (85.2% and 65.8%, respectively) resembled more closely the frequencies in American and European cohorts, rather than the Indian population, which is in agreement with our population genetics data where we see a cline passing through Pakistan and diminishing in intensity toward India [17-19]. The frequency of the G-allele of rs3825942 in Indian patients and controls (92.3% and 74.2%, respectively) was also somewhat lower than in our Pakistani cohorts (97.5% in PEXG and 83.9% in controls), and the OR was slightly lower in the Indian cohort (4.17) compared to our Pakistani cohort (6.98). The lower association of rs3825842 in the Indian and Pakistani populations compared to other populations from America, Europe and Asia (overall OR 10.89), suggests that additional genetic factors may play a role in PEXG in India and Pakistan, either within the *LOXL1* or in other genes. This is supported by a recent study demonstrating that neither rs1048661 (R141L) nor rs3825942 (G153D) affect the amine oxidase activity of LOXL1, suggesting that other unknown genetic factors or molecular mechanisms may be more relevant to the development of PEXG [20].

In previous studies we have identified significant associations with polymorphisms in the glutathione S-transferase (*GSTT1* and *GSTM1*) [21] and tumor necrosis factor alpha (*TNF-α*) [12] with PEXG. Among these higher OR was observed for *TNF-α* (8.37) as compared to *LOXL1* (6.98), *GSTT1* (4.26), and *GSTM1* (3.81), the combined effect of all these genes probably have a significant contribution in causing PEXG in the Pakistani cohort. The present study confirms the involvement of the two *LOXL1* coding polymorphisms in PEXG in the Pakistani population. Further studies at the functional level are required to better understand the involvement of *LOXL1*, glutathione S-transferase and tumor necrosis factor alpha in PEXG, which could potentially aid the development of therapeutic approaches for the disease.
